# Antioxidant and Anti-Tumor Effects of Dietary Vitamins A, C, and E

**DOI:** 10.3390/antiox12030632

**Published:** 2023-03-03

**Authors:** Alexander J. Didier, Jennifer Stiene, Lauren Fang, Dean Watkins, Lance D. Dworkin, Justin F. Creeden

**Affiliations:** Department of Medicine, The University of Toledo College of Medicine and Life Sciences, Toledo, OH 43614, USA

**Keywords:** antioxidants, reactive oxygen species, oxidative stress, vitamins, neoplasms, carcinogenesis

## Abstract

Oxidative stress, a condition characterized by an imbalance between pro-oxidant molecules and antioxidant defense systems, is increasingly recognized as a key contributor to cancer development. This is because the reactive oxygen species (ROS) generated during oxidative stress can damage DNA, proteins, and lipids to facilitate mutations and other cellular changes that promote cancer growth. Antioxidant supplementation is a potential strategy for decreasing cancer incidence; by reducing oxidative stress, DNA damage and other deleterious cellular changes may be attenuated. Several clinical trials have been conducted to investigate the role of antioxidant supplements in cancer prevention. Some studies have found that antioxidant supplements, such as vitamin A, vitamin C, and vitamin E, can reduce the risk of certain types of cancer. On the other hand, some studies posit an increased risk of cancer with antioxidant supplement use. In this review, we will provide an overview of the current understanding of the role of oxidative stress in cancer formation, as well as the potential benefits of antioxidant supplementation in cancer prevention. Additionally, we will discuss both preclinical and clinical studies highlighting the potentials and limitations of preventive antioxidant strategies.

## 1. Introduction

Cancer is a leading cause of death globally, accounting for approximately 10 million deaths worldwide. The global burden of cancer continues to increase, with estimates predicting 28.4 million cases in 2040, a 47% rise from 2020 which is largely due to population growth and aging [1]. Although age plays a role in carcinogenesis, the development of cancer is a complex process that involves genetic mutations, environmental factors, and lifestyle choices. One key contributor to cancer development is oxidative stress, a condition characterized by an imbalance between pro-oxidant molecules and antioxidant defense systems. Antioxidant supplementation has been proposed as one strategy to reduce oxidative stress and thus carcinogenesis, with vitamins A, C, and E at the forefront of discussion.

**Figure 1:**
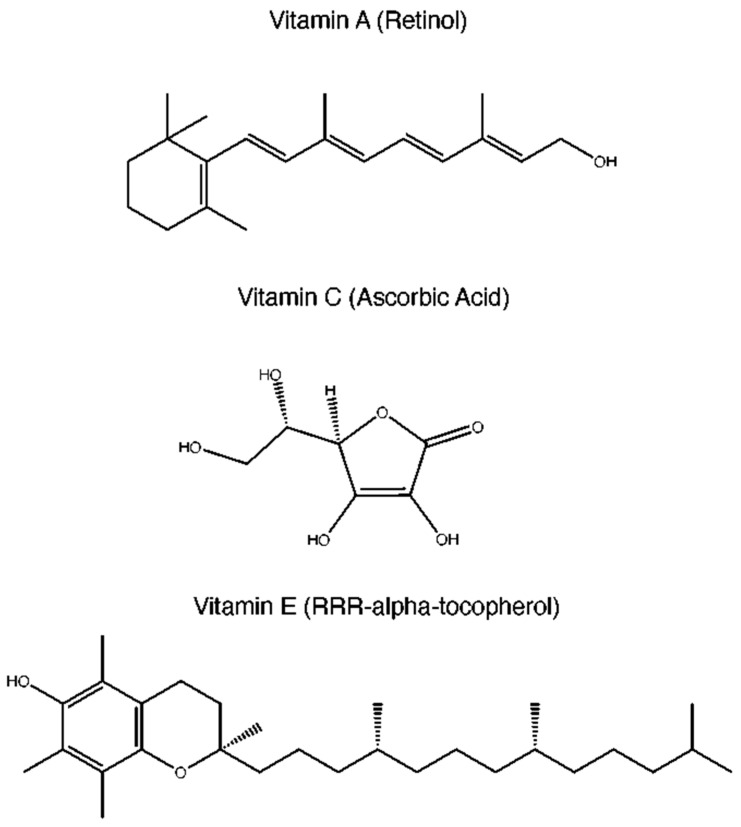
Chemical structures of vitamins A, C, and E.

Reactive oxygen species (ROS) are generated as a byproduct of normal cellular metabolism and can cause damage to DNA, proteins, and lipids if their levels become too high. This can lead to mutations and other cellular changes that promote cancer growth when ROS production outpaces the production of antioxidants, whose role is to neutralize ROS. This is termed oxidative stress. ROS are produced during a number of physiologic processes, such as mitochondrial oxidative metabolism and cellular response to bacterial invasion [2]. Oxidative stress can lead to tumor initiation via multiple mechanisms, including disruption of vital biochemical pathways and oxidation of nuclear DNA which leads to genetic mutations and instability [2]. In response to increased ROS, the body can upregulate the production of antioxidant molecules such as catalase, superoxide dismutase (SOD), glutathione (GSH), or it can upregulate non-enzymatic antioxidants. Non-enzymatic antioxidants are composed of two subgroups: non-enzymatic metabolic antioxidants and non-enzymatic nutrient antioxidants. Non-enzymatic metabolic antioxidants include bilirubin, melatonin, uric acid, coenzyme Q10, and GSH. Non-enzymatic antioxidants, which include vitamin A, vitamin C, and vitamin E (Figure 1), are commonly used dietary supplements for general health purposes. Given their safe profile and potential link with a decreased risk of cancer, they represent an attractive option as preventive anti-cancer agents.

In this review, we will provide an overview of the current understanding of the role of oxidative stress in cancer formation, as well as the potential benefits of antioxidant supplementation in cancer prevention. Additionally, we will discuss both preclinical and clinical studies, highlighting the promise and limitations for this potential preventive strategy.

## 2. Rationale for Antioxidant Use in Cancer Prevention

### 2.1. Oxidative Stress and Carcinogenesis

The association between ROS and carcinogenesis is well recognized [3,4]. ROS is a broad term encompassing oxygen derivatives that have accepted free electrons, including hydrogen peroxide (H_2_O_2_), superoxide (O_2_^−^), and hydroxyl (OH^−^). Free electrons can then be used to oxidize other molecules, including nucleic acids, lipids, or proteins [5]. Oxidization of any of these molecules can lead to a disruption of their normal function, with subsequent downstream effects. ROS may be created from either endogenous sources, such as mitochondrial reactions [6], or exogenous sources, such as cigarette smoke or ionizing radiation [2]. These ROS then create a state of oxidative stress, where the balance between ROS and their counterpart antioxidants is disturbed in favor of the oxidants [7]. Antioxidants such as glutathione peroxidase, catalase, and superoxide dismutase serve as sentinels, protecting cells from the potentially harmful effects of ROS. If there is an overwhelming increase in ROS, as in oxidative stress, the balance shifts in a manner that results in cell growth and chromosomal instability, promoting tumor development [5]. In addition, ROS can also interfere with normal processes such as signal transduction, protein synthesis, and cell division, which can further increase the risk of cancer [8].

**Figure 2:**
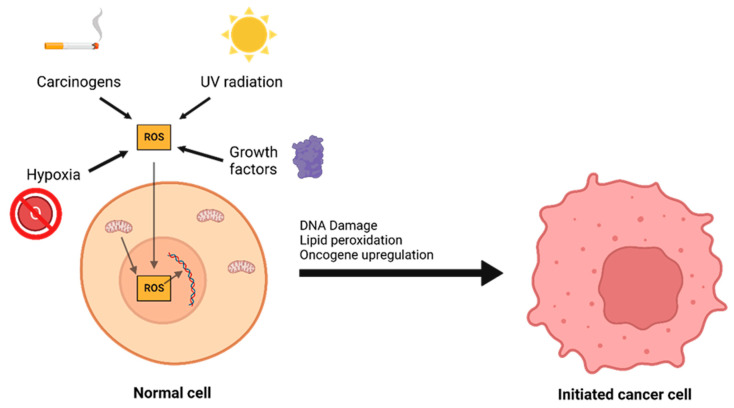
A number of environmental and chemical factors, including carcinogens, UV radiation, hypoxia, growth, and mitochondrial dysfunction can contribute to the formation of reactive oxygen species (ROS). ROS can then oxidize DNA and lipids or lead to oncogene upregulation, causing a normal cell to transform into an initiated cancer cell.

The interplay between ROS and antioxidants is of critical importance in the development of cancer. There are three distinct phases to tumor formation: initiation, promotion, and progression [9]. In the initiation phase, a normal cell sustains a mutation to its genomic DNA, becoming an initiated cell. Multiple mechanisms for this have been previously described, including interaction with physical carcinogens such as UV light or with chemical carcinogens that directly damage DNA. Additionally, spontaneous mutations may occur after DNA is repaired incorrectly, which can promote the formation of initiated cells. Oxidative stress is a major contributor to spontaneous DNA mutations [10] (Figure 2). After the initiation phase, the mutated cell reaches the promotion phase, where it selectively proliferates and forms a preneoplastic lesion. Finally, cancer progression occurs, during which the lesion replicates and cells within the tumor population begin to accrue mutations. These mutations may confer selective advantages, such as increased production of growth factors, and become dominant within the population—a process referred to as clonal selection.

Oxidative stress has been shown to impact both oncogenes and tumor suppressor genes, relationships which are largely modulated via a master regulator called nuclear factor erythroid 2-related factor 2 (Nrf2) [11]. Nrf2 and its repressor protein kelch like ECH associated protein 1 (Keap1) tightly regulate levels of ROS by modulating a number of pathways, including the antioxidant response element pathway [12,13]. Nrf2 positively regulates ARE to increase the expression of antioxidant enzymes, such as heme oxygenase 1 (HO-1) and NAD(P)H:quinine oxidoreductase 1 (NQO1) [14]. In mice who received Protandim^®^ (LifeVantage Corp., Lehi, UT, USA), a dietary supplement consisting of five Nrf2 activators, skin tumor incidence and multiplicity (the number of induced tumors) were reduced by 33% and 57%, respectively, compared to mice on a control diet [15]. These results are hypothesized to stem from the role of Nrf2 in modulating oxidative stress by inducing the formation of endogenous antioxidant enzymes, which may regulate p53 mitochondrial translocation [16]. Additionally, Nrf2 is regulated by tumor suppressor gene breast cancer gene 1 (BRCA1). The BRCA1 caretaker gene plays a role in DNA repair and homologous recombination. Through its interactions with Nrf2, BRCA1 upregulates several genes responsible for antioxidant generation, further highlighting the necessary relationship between tumor suppressor genes and oxidative stress [17]. Somatic mutations interrupting the interaction between Nrf2 and Keap1 have been identified in some cancer patients, including lung and skin cancer [18,19,20]. Further, the oncogenic Ras pathway has been shown to upregulate oxidative stress independently from Nrf2 [21]. Point mutations in Ras codons 12, 13, or 61, or one of the three Ras genes (H-Ras, K-Ras, and N-Ras) activate these oncogenes [22]. In mouse lung epithelial cells, upregulation of human mutant K-ras led to an increase in COX2-generated ROS and single-stranded DNA breaks. In turn, this resulted in significant oxidative stress which may contribute to carcinogenesis. Activating Ras mutations have been discovered in approximately 30% of human tumors, including up to 90% of pancreatic adenocarcinomas, demonstrating its role in tumor development [22].

### 2.2. The Role of Antioxidants: Protection and Paradox

ROS have been described to cause damage to proteins in various signaling pathways, which may be related to their carcinogenic properties [23]. ROS have been implicated to cause aberrant activity of the mitogen-activated protein kinase (MAPK) family of proteins, which are necessary for cell cycle arrest or progression [24]. One protein in the MAPK family, extracellular signal regulated kinase 1 and 2, (ERK1/2), may interact with ROS. ROS have been described to inactivate phosphatases that dephosphorylate ERK1/2, leading to continued ERK activation and cell proliferation [25]. Similarly, hydrogen peroxide and mitochondrial ROS can inactivate the PTEN tumor suppressor gene, leading to uncontrolled cellular proliferation [26]. Finally, ROS has been shown to interact with the hypoxia inducible factor (HIF) family of transcription factors. ROS stabilize the HIF proteins during periods of hypoxia, leading to increased transcription [27]. This has been demonstrated by Chi Dang et al., who showed that the addition of antioxidants vitamin C and N-acetylcysteine resulted in inhibition of carcinogenesis due to decreased HIF activity, illustrating the role that ROS plays in interacting with proteins [27].

Further, ROS can damage lipids through a process called lipid peroxidation, where polyunsaturated fatty acid (PUFA) side chains of lipids are autoxidized via a free radical reaction [27]. During this reaction, a large number of lipid hydroperoxides are produced via a chain-propagating reaction [27]. Additionally, the lipid peroxyl radical formed during this reaction can form a cyclic peroxide, which further decomposes into a number of breakdown products, including malondialdehyde (MDA) and 4-hydroxy-2-nonenal (4-HNE). Both MDA and 4-HNE are significant because of their mutagenicity, which can lead to DNA damage [28]. Specifically, MDA reacts with nucleosides deoxy-guanosine and cytidine, where it forms DNA adducts that then lead to the development of a pyrimidopurinone called pyrimido [1,2-a]purin-10(3H-)one (M1G or M1dG) [29]. M1dG levels have been shown to correlate with higher HIC1 (a tumor suppressor gene) methylation levels in tobacco smokers, which may result in increased carcinogenesis [30]. Similarly, 4-HNE has been shown to play an important pathological role in carcinogenesis via interaction with mitochondria [31]. 4-HNE may also promote breast cancer growth and angiogenesis through interactions with HIF, lending support to its tumorigenic properties [32].

Antioxidants, which may be produced endogenously or exogenously, protect our cells from the harmful effects of ROS by donating their own electrons in order to neutralize free radicals. Interestingly, cancer cells themselves may develop mutations that upregulate their development of antioxidants, providing evidence of an evolutionary counterbalance system that allows tumor cells to survive in states of high oxidative stress [33]. Antioxidant manipulation by neoplastic cells presents a paradox in which our natural defense system is hijacked to promote carcinogenesis.

Endogenous antioxidants, produced by the body itself, include peroxidase enzymes such as glutathione peroxidase (GPX), transferases such as glutathione S-transferase (GST), and superoxide dismutase (SOD). These enzyme groups protect against the initial stages of carcinogenesis by neutralizing ROS-induced DNA damage. GST isoforms have been shown to block the formation of liver and colon neoplasms in mice after exposure to carcinogens [34,35]. The antioxidant mechanism of GST involves modulating downstream effector pathways to prevent the formation of ROS [36]. However, GSTs have also been implicated in oncogenic processes, including activation of signaling proteins such as Akt [37]. A similar phenomenon can be observed with GPX enzymes. GPX has been shown to prevent colorectal carcinogenesis, potentially through inhibiting inflammation and DNA damage [38]. In fact, loss of GPX in mutant mice lead to an increase in the number and aggressiveness of tumors, which suggests that GPX loss may have a preventive role in cancer progression as well as initiation [38]. However, GPX may also contribute to tumorigenesis—GPX knockout mice exhibited decreased colon tumor development after treatment with azoxymethane to induce inflammation-triggered carcinogenesis, suggesting a promotive role for GPX in the tumor microenvironment potentially leading to tumor formation [39]. Finally, SODs catalyze the breakdown of the superoxide anion into oxygen and hydrogen peroxide [40]. Reduced activity of SOD molecules has been reported in cancer cells, and individuals with low SOD expression may be predisposed to cancer development [41,42,43,44,45]. Of note, pancreatic cancer invasion and migration is promoted by SOD via hydrogen peroxide mediated NF-kB and ERK activation [46,47]. Additionally, metastatic pancreatic tumors tend to increase SOD expression, which may improve their ability to survive in a new tumor microenvironment [48]. Thus, the pro-tumor and anti-tumor roles of endogenous antioxidants sometimes appear contradictory, likely due to incompletely elucidated protein interactions within each system, warranting further investigation. These contradictory roles may also be due to site-specific effects of ROS. In a meta-analysis of 21 studies including 2121 patients, Qing and colleagues discovered that increased levels of 8-hydroxy-2′-deoxyguanosine (8-OHdG), a product of oxidative stress, was a significant marker of poor survival for cancer patients [49]. However, the opposite effect was noted in breast cancer, with low 8-OHdG levels associated with worse prognoses. This is thought to be due to the upregulation of Nrf2 that occurs in breast cancer cells, which may promote breast cancer progression and aggressiveness [50]. These results indicate a potential site-specific effect, where oxidative stress may be carcinogenic in one site, but protective at another.

### 2.3. A, C, and E Antioxidants Specifically

Exogenous antioxidants are those which our bodies cannot produce themselves, including vitamins A (and the related family of carotenoid molecules), C (ascorbate), and E [51]. Vitamin A is structurally related to β-carotene (a pro-vitamin A compound) [52], and is composed of two subgroups, retinol (Vitamin A_1_) and dehydroretinol (Vitamin A_2_). These molecules differ in their antioxidant mechanisms. Vitamin A, which refers to the larger family of vitamins, can combine with peroxyl radicals, acting as a chain-breaking antioxidant before the peroxyl radicals can interact with lipids and generate hydroperoxides, thus preventing cellular damage [53]. Carotenoids can scavenge singlet oxygen and peroxyl radicals, both of which are highly reactive and unstable [54]. Additionally, carotenoids may exert indirect antioxidant activity by upregulating SOD and catalase [55]. Vitamin C is a common exogenous supplement that can scavenge free radicals and has a well-established protective role in carcinogenesis [56]. Vitamin C is maintained in its reduced form by interacting with glutathione, allowing it to reduce and neutralize ROS [57,58]. Importantly, vitamin C is capable of regenerating vitamin E in lipid membranes by using reducing equivalents and glutathione. Vitamin E collectively describes a group of related tocophenols and tocopherols [59]. Of these, α-tocopherol has been highly studied due to its high bioavailability [60]. This lipid-soluble antioxidant protects lipid membrane oxidation by reacting with lipid radicals that are produced in the lipid peroxidation chain reaction [61]. This reaction removes the free radicals, preventing the peroxidation reaction from continuing and damaging cell membranes. During this reaction, oxidized α-tocopherol is generated, which interacts with ascorbates who reduce the oxidized α-tocopherol and recycle it back to its antioxidant form [62]. Additionally, retinol may also interact with tocopheroxyl radicals and regenerate α-tocopherol [53].

### 2.4. The Link between Oxidative Stress and Cancer: Real World Evidence

While there is a strong body of evidence to suggest a link between oxidative stress and carcinogenesis at the cellular level, an epidemiological link is less clear-cut. A number of published observational studies have evaluated the relationship between increased oxidative stress and cancer incidence, yielding mixed results. Rossner et al. assessed the role of oxidative stress and breast cancer by measuring urinary levels of two oxidative stress biomarkers, 15-F(2t)-isoprostane (15-F(2t)-IsoP) and 8-oxodeoxyguanosine (8-oxodG), in 400 cases and 401 controls [63]. They found a statistically significant trend in breast cancer risk with increasing quartiles of 15-F(2t)-IsoP levels. When the highest quartile of 15-F(2t)-IsoP was compared with the lowest quartile, a 1.88 times increased risk of breast cancer was determined (95% CI: 1.23–2.88, *p*(trend) = 0.002). These results were consistent when controlled for causes of oxidative stress, including alcohol, smoking, obesity, and age. However, these findings conflict with those of Dai and colleagues, who found no significant differences in urinary excretion of isoprostanes in a similarly designed study [64]. Interestingly, the Dai study revealed that higher levels of urinary isoprostanes in obese women strongly correlated with breast cancer development but were associated with reduced risk among non-obese women. The Dai study only examined urinary levels of 15-F(2t)-IsoP, not circulating levels or levels within the breast, making it difficult to draw robust causative conclusions; it is impossible to determine whether the increased levels of ROS led to cancer, or whether the cancer led to increased levels of ROS. Still, this suggests some multifactorial relationship between ROS and carcinogenesis.

Additionally, there may be temporal-dependent effects of ROS on carcinogenesis [65]. Given that cancer cells develop mutations which allow them to upregulate the production of antioxidants, it is possible that there may be a stage-dependent effect of oxidative stress—oxidative stress is needed for an inciting event to catalyze the transformation of a normal cell to a neoplastic cell, and then over time the oxidative stress decreases as the transformed cell adapts to the stress. This theory is supported by O’Farrell and colleagues, who demonstrated that in the progression of Barrett’s esophagus to esophageal adenocarcinoma, 8-oxodG and 4-HNE biomarkers decreased across each progressive malignant transformation [66]. However, observational studies are limited in their ability to assess the temporal relationship of ROS in cancer development. In one systematic review of oxidative stress biomarkers in prostate cancer, 21 of 23 studies reported at least one marker of oxidative stress to be higher in men with prostate cancer [65]. These results align with those of Barocas et al. and Brys et al., who demonstrated that increased levels of markers of oxidative stress were strongly associated with the occurrence of prostate cancer [67,68]. While these demonstrate some evidence that a relationship exists between oxidative stress and cancer, it is important to note that biomarkers were collected after patients had already been diagnosed with cancer; thus, it is impossible to determine the temporal sequence between oxidative stress and prostate cancer. Further, as discussed previously, there may be site-specific effects occurring that allow oxidative stress to promote tumor development. Due to the potential relationship between oxidative stress and carcinogenesis, antioxidant vitamin supplementation has been proposed as a potential prophylactic strategy due to their low cost and high safety profile.

## 3. Vitamin A

### 3.1. Source and Forms

Strictly speaking, vitamin A is all-*trans*-retinol [69]. However, vitamin A, as used in this review, refers to two general groups of compounds with physiologic vitamin A activity: vitamin A precursor carotenoids and non-carotenoid vitamin A precursors and metabolites [70]. Examples of vitamin A precursor carotenoids include alpha and beta carotene. Non-carotenoid vitamin A precursors and metabolites include retinol esters (inactive storage form), retinal (used for vision), and retinoic acid (potent transcription factor) [69,71]. Diet is the main source of vitamin A, which includes preformed vitamin A and provitamin A. Preformed vitamin A, as the name suggests, has already been converted by organisms lower in the food chain. Therefore, animal-derived foods are a source of preformed vitamin A (retinol, retinal, retinoic acid, and retinyl esters). Provitamin A consists of plant-derived carotenoids such as beta-carotene alpha-carotene, and beta-cryptoxanthin. However, not all carotenoids possess provitamin A activity. Of the 600 types of carotenoids, approximately 50 have provitamin A activity [71]. Most vitamin A supplements contain a combination of provitamin A and preformed vitamin A.

### 3.2. Antioxidant Activity of Vitamin A

**Figure 3:**
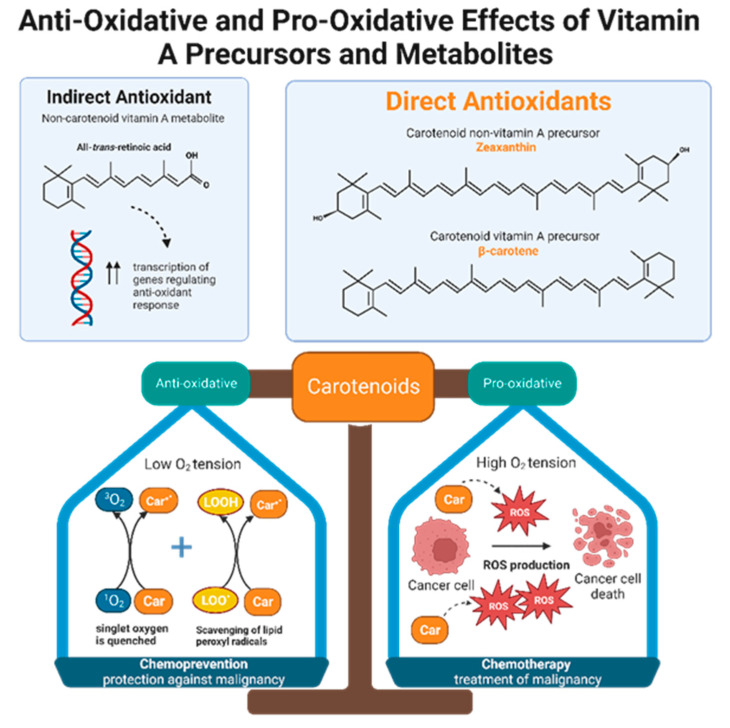
Non-carotenoid vitamin A precursors and metabolites include retinol, retinal, retinyl esters, and retinoic acid. Specifically, all-trans-retinoic acid demonstrates indirect antioxidant activity by increasing expression of gene products that enhance cellular response to oxidative stress. Carotenoids include both non-vitamin A and provitamin A precursors. Carotenoids, regardless of their vitamin A activity, exhibit direct anti-oxidizing abilities via radical scavenging and singlet oxygen quenching. Furthermore, carotenoids are dynamic agents that can produce either an anti-oxidative or pro-oxidative response, depending on the partial pressure of oxygen in the cellular environment. Malignant cells maintain high intracellular ROS levels, creating high oxygen tension. Under this condition, carotenoids exert a pro-oxidative response by increasing ROS production to generate oxidative stress that will kill cancer cells. This suggests that carotenoids have potential chemotherapeutic effects. In normal, non-neoplastic cells, carotenoids balance the production and elimination of ROS. This suggests that carotenoids may have a role in chemoprevention.

Retinol inhibits peroxidation of liposomes and fatty acid esters in vitro, making it an effective peroxyl radical scavenger [71]. Compared to tocopherol, retinol’s scavenging ability is even greater, but only when radical species originate from the lipid bilayer and not from the aqueous environment [71]. Like retinols, carotenoids can also scavenge peroxyl radicals, and they can quench singlet oxygen, a partially reduced oxygen that is highly reactive and unstable. The antioxidant potencies of preformed vitamin A versus carotenoids have been compared via in vitro studies of liposomal systems. Carotenoids with at least 11 conjugated double bonds (beta-carotene, lutein, lycopene, cryptoxanthin, and zeaxanthin) are five times more effective than retinoids such as retinol, retinol palmitate, and retinoid acid in resisting oxidation [71]. Alpha and beta carotene, lycopene, lutein, and cryptoxanthin constitute the majority of carotenoids present in human plasma. Despite the fact that lutein and lycopene have little to no provitamin A activity, they demonstrate significant antioxidant effects and may be even more potent antioxidants than provitamin A carotenoids [71]. In other words, it is not necessarily the ability to form vitamin A that gives some carotenoids greater antioxidant potential than others, but rather the intrinsic properties of carotenoids themselves. Interestingly, carotenoid antioxidant activity is greatest at physiologic oxygen tension and becomes less protective in a concentration dependent manner as oxygen tension increases [71,72]. Therefore, depending on physiological conditions, antioxidant carotenoids can become pro-oxidant [72]. One possible theory that emerges from this observation is that carotenoids may function as both chemopreventive and chemotherapeutic agents, depending on the cellular environment (Figure 3). Neoplastic cells maintain high intracellular ROS levels, and it is thought that under these conditions, the pro-oxidant activities of carotenoids predominate over their antioxidant activities [73]. Carotenoids appear to take advantage of the higher ROS levels in malignant cells to exert greater oxidative stress that will enhance cancer cell apoptosis [74]. In contrast, within the milieu of normal cells, carotenoids optimize oxidative balance by adopting double duties of ROS scavenging and ROS production [74].

Much of the literature has focused on the antioxidative properties of carotenoids. This is because the current consensus is that only carotenoids possess direct antioxidative effects, while non-carotenoid vitamin A precursors and metabolites do not. In fact, associating “vitamin A” with “antioxidant” is arguably misleading and inaccurate [69]. It is known that, in vivo, fat soluble vitamin A compounds such as retinol, retinal, and retinoic acid are attached to vitamin A-binding proteins and are highly protected from the aqueous environment [69]. Transport and sequestration is also highly regulated. Many suggest that with limited accessibility physiologically, vitamin A cannot serve as a strong antioxidant with the ability to directly quench or generate radicals [69]. Rather, non-carotenoid vitamin A precursors and metabolites may serve more of an indirect role in reducing oxidative stress as transcription regulators [69]. For instance, all-trans-retinoic acid (ATRA) is a non-carotenoid vitamin A metabolite that binds to transcription factors to regulate gene expression and cellular differentiation. Upon binding to its nuclear receptors, ATRA activates TRX, GCLC, and GCLM gene expression. TRX regulates the thioredoxin antioxidant system in the cell cytoplasm, which is responsible for protein thiol maintenance and hydroperoxide reduction [69]. GCLC and GCLM control the production and regeneration of the antioxidant glutathione [69]. By increasing the expression of genes that facilitate responses to oxidative stress, ATRA can produce an indirect anti-oxidative effect [69].

In vitro and in vivo studies demonstrate that beta carotene has a protective effect against cancer [75]. Studies of mouse mammary cell organ cultures treated with chemical carcinogens to induce malignant transformation demonstrated that the addition of beta-carotene had a significant effect in preventing neoplastic changes [76]. Beta-carotene has also been shown to block genotoxic compound production by Chinese hamster ovary cells with chromosomal aberrations [76]. Both dietary and injected beta-carotene have demonstrated efficacy as a preventive agent for UV-induced skin tumors in mice [76]. Beta-carotene administration resulted in decreased skin tumor incidence in mice, but this only occurred at certain concentrations. Namely, BC doses of 700 mg/kg were protective; however, a 10-fold decrease in BC dose did not reduce the number of tumors formed [76]. In addition, BC has a role in preventing oropharyngeal tumors in mice. Topical BC has been shown to inhibit squamous cell carcinomas in the cheek pouches of hamsters, while dietary BC has demonstrated decreased incidence of malignant salivary gland tumors [76]. Regarding GI tumors, there is a reduced incidence of colon cancer in rats treated with low-dose dietary BC [76]. Retinoids have been tested as cancer chemoprevention agents in a number of in vivo studies evaluating skin, respiratory tract, GI, breast, and urinary bladder tumor models [77]. However, the same retinoid may have different effects depending on the organ. For instance, 13-*cis*-retinoic acid inhibits carcinogen-induced urinary bladder cancer in rats and mice but shows no effect against mammary cancer in the rat [77]. As another example, retinyl acetate exhibits efficacy in the rat mammary cancer model but provides minimal chemopreventive protection against skin and mammary tumors in mice [77]. In addition to the many in vivo studies demonstrating that vitamin A precursors exhibit protective effects against cancer, several experiments also point to their potential antioxidant mechanism. Superoxide, a type of ROS, can oxidize hemoglobin and damage RBC membrane phospholipids, causing accumulation of phospholipid hydroperoxides. In a study by Nakagawa et al., mice were fed all-trans BC at 6 g/kg or 30 g/kg for 1 week in addition to their regular semi-synthetic diet [78]. Compared to the control group, BC diet supplementation significantly prevented the accumulation of phospholipid hydroperoxides in mouse RBCs. However, this strong antioxidative effect was only observed in the RBCs and not in the plasma, liver, or lungs [78]. This suggests that vitamin A derived from BC may selectively target certain tissues or cells to exert its antioxidant effects. Of note, the standard semi-synthetic diet across all groups contained 50 mg alpha-tocopherol/kg, and there was no control group studying the effects of an all-trans BC diet alone. Therefore, it is impossible to ignore any potential confounding effects of vitamin E on vitamin A and anti-phospholipid hydroperoxidation.

### 3.3. Observational Studies of Vitamin A Supplementation and Cancer Prevention

Observational studies of vitamin A supplementation for cancer chemoprevention have demonstrated mixed results, showing either reduced or unchanged risk of cancer development [70]. However, after the results of two large, randomized clinical trials pointed to increased risk for lung cancer development following BC supplementation, no further trials of vitamin A for chemoprevention have been initiated [79].

The most recent clinical trial investigating vitamin A chemoprevention was the Alpha-Tocopherol, Beta-Carotene (ATBC) trial, which studied males aged 50–69 years who smoked an average of 20.4 cigarettes a day for an average of 35.9 years. Participants took a daily supplement of BC (20 mg/day), with or without vitamin E (50 mg/day) for 5 to 8 years. BC supplementation was found to be associated with an 18% increased risk of lung cancer [51]. Additionally, the all-cause mortality of participants undergoing BC supplementation was 8% higher, with most deaths occurring due to lung cancer or ischemic heart disease [51]. A follow-up study tracked the ATBC participants for an additional 18 years when they had stopped taking BC supplements. Although most continued to smoke, the original BC subjects did not have an elevated risk of lung cancer. Instead, they were found to have a 20% higher risk of death secondary to prostate cancer [80]. Before ATBC, the Carotene and Retinol Efficacy Trial (CARET) was the earliest investigation of vitamin A supplementation effect on lung cancer prevention [81]. CARET participants included individuals with known increased risk for lung cancer, such as current and former smokers with a minimum 20 pack-year history as well as men with occupational exposure to asbestos. Subjects took two different vitamin A supplements, BC (30 mg/day) plus retinyl palmitate (25,000 IU/day). The trial was stopped prematurely after 4 years due to an unexpected 28% increase in lung cancer risk, a 46% increase in death from lung cancer, and an increased all-cause mortality of 17% [81]. Subsequent evaluation of CARET participants 6 years later when they had stopped taking supplements did not reveal any significant differences in lung cancer risk between the experimental and control groups. However, one exception was noted, which was that women who were originally assigned to take the two vitamin A supplements had a 33% higher risk of lung cancer [82].

Two randomized clinical trials of vitamin A chemoprevention, one for skin cancer and another for squamous cell cancer of the head and neck, did not reveal significantly reduced rates of new cancer occurrence. When patients with a recent non-melanoma skin cancer were assigned to either 50 mg of beta carotene or placebo daily with both groups undergoing annual skin examinations, 5-year follow up data indicated no difference in the rate or mean number of new squamous or basal cell carcinomas developed [83]. In another study by Hong et al., disease-free patients who had received previous treatment for squamous cell carcinoma of the larynx, pharynx, or oral cavity were randomly assigned to 13-*cis*-retinoic acid (50–100 mg/m^2^ of body surface area) or placebo daily [84]. There were no significant differences in primary cancer recurrence. However, the 13-*cis*-retinoic acid group demonstrated significantly fewer second primary tumors (*p* = 0.005), defined as tumors presenting at a different site than the original tumor. Therefore, the investigators concluded that daily treatment with high dose 13-*cis*-retinoic acid was effective in preventing second primary tumors but not recurrences of the original tumor [84].

Overall, cancer rates do not seem to be influenced by vitamin A supplementation, according to data published from randomized, double-blind, placebo-controlled trials. For example, one study assigned male physicians with current, former, and non-smoking status to one of four groups: 325 mg aspirin on alternate days plus beta carotene placebo, 50 mg beta carotene on alternate days plus aspirin placebo, both active agents, or both placebos [85]. The incidence of malignancy, overall mortality, and cardiovascular disease was not significantly different after 12 years of treatment and follow-up. In particular, the incidence of cancer of the lung, colon, rectum, prostate, stomach, pancreas, and brain was comparable between the beta carotene and placebo groups. This was true for malignant melanoma, leukemia, and lymphoma as well [85]. The Women’s Health Study was another trial testing aspirin, vitamin E, and beta carotene as possible cancer prevention agents [86]. Women aged 45 years or older were included, and the beta-carotene dose was 50 mg on alternate days. The findings were similar to those of the Physicians’ Health Study conducted by Hennekens et al. No statistically significant differences in cancer incidence were identified for sites including the breast, uterus, cervix, ovary, stomach, colon, rectum, thyroid, bladder, brain, and pancreas. The incidences of melanoma, leukemia, and lymphoma were similar between treatment and control groups. Of note, the beta-carotene component of the intervention was terminated early after a median treatment duration of 2.1 years due to the null findings on beta carotene and cancer incidence in the Physicians’ Health Study as well as concern over the increased harm in patients with elevated lung cancer risk [86].

Despite its detrimental effects in individuals at high risk of developing lung cancer and lack of cancer protection in other studies, vitamin A may still play a prophylactic role in certain malignancies. Natural and synthetic forms of vitamin A have been studied for oral cavity and oropharyngeal premalignant lesions. Garewal et al. conducted a multicenter, double-blind, placebo-controlled trial, evaluating 50 subjects with documented precancerous leukoplakia [87]. These subjects were given BC 60 mg/day for 6 months. Responders to BC supplementation were then randomized to continue BC or placebo therapy for an additional 12 months. By 6 months, 26 participants (52%) had demonstrated a clinical response. Only 2 (18%) of 11 in the continued BC cohort and 2 (17%) of 12 in the placebo group relapsed. Similar results were achieved by Sankaranarayanan et al., who conducted a randomized, double-blind placebo-controlled trial of 160 people in India with oral precancerous lesions, assigning them treatment with vitamin A (300,000 international units per week), BC (360 mg per week), or placebo over a 1-year period [88]. The complete regression rates were 52%, 33%, and 10% in the vitamin A, BC, and placebo groups, respectively. Relapse was observed in 50–66% of participants who had previously responded to either BC or vitamin A when they discontinued supplementation.

Some clinical studies support the notion that the preventive action of vitamin A is highly selective for certain malignancies, such as oral cancer. However, others have indicated no significant protective effects of vitamin A for any cancer type. Many studies have combined vitamin A with other antioxidants and/or micronutrients as part of the intervention; however, this makes it difficult to determine the isolated effects of vitamin A [89,90]. Additionally, large studies of vitamin A chemoprevention in relatively vitamin A deficient patient populations outside of the US raise concern that the seemingly protective effects of supplementation may be due more to addressing deficiency rather than true antioxidant activity [89,90]. Various forms of vitamin A, both preformed and provitamin, have been used in clinical studies with different dosages. Comparisons between such studies are challenging.

### 3.4. Safety Profile of Vitamin A

Vitamin A is generally considered a safe compound in the diet. Provitamin A compounds are highly regulated by feedback mechanisms, as they must be cleaved to retinal before absorption. This is a step dependent on vitamin A status, which is the body’s nutritional adequacy at the time of ingestion. In contrast to provitamin A, preformed vitamin A is not well controlled by feedback regulation and therefore can accumulate to toxic concentrations [79]. However, vitamin A toxicity is not always apparent or detectable as an increased serum concentration. This is because circulating vitamin A levels do not consistently reflect storage amounts in the liver and adipose tissue, where vitamin A reserves are maintained in the form of long chain fatty acid esters such as retinyl palmitate, oleate, myristate, and linoleate [70,71,79]. Most cases of chronic vitamin A toxicity are attributable to the long-term ingestion of large quantities of synthetic, preformed vitamin A in doses higher than 10 times the recommended daily allowance (RDA) [79]. In adults, chronic toxicity is roughly equivalent to long-term ingestion of approximately 33,000 international units (10,000 μg) of retinol [79]. Acute vitamin A toxicity in adults is also possible and occurs with a single ingested dose of vitamin A > 660,000 international units (>200,000 μg) [79]. Manifestations of acute vitamin A toxicity include dry lips, cheilitis, or dry mucosa, whereas chronic vitamin A toxicity may lead to bone spurs, central nervous system dysfunction, or renal dysfunction [91]. These symptoms typically reverse with discontinuation of vitamin A; however, the adverse effects on the central nervous system and kidneys may be irreversible if ingestion is continued [91]. The Food and Nutrition Board (FNB) at the National Academies of Sciences, Engineering, and Medicine advises against the use of beta-carotene supplements for the general population, except to prevent vitamin A deficiency [70].

## 4. Vitamin C

### 4.1. Antioxidant Effects of Vitamin C

**Figure 4:**
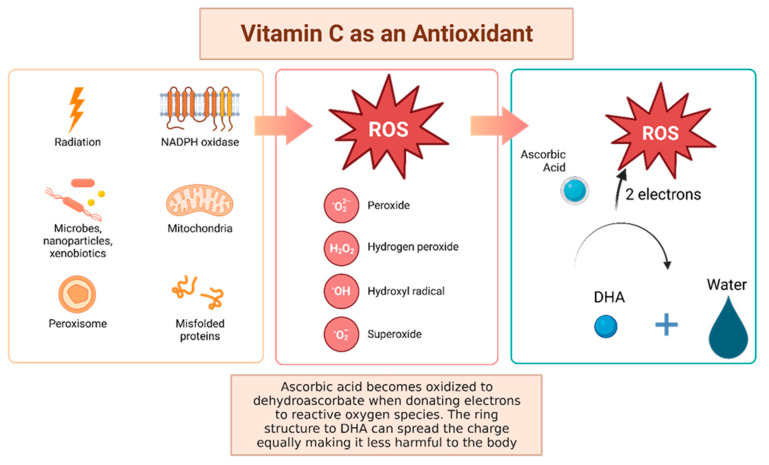
Vitamin C has been shown to have antioxidant properties, allowing it to reduce free radicals that may cause harmful damage to DNA. Reactive oxygen species (ROS) may be made by peroxisomes, radiation, the mitochondria, and more biological processes which result in ROS. Vitamin C acid, when ingested, contains electrons that it can give to reactive oxygen species. These will be reduced to water, and therefore will not be harmful to the body [92]. The oxidized version of vitamin C, or dehydroascorbate, has the ability he ability to even out the positive charge with its ring structure ensuring that it, itself, is not going to damage cells [93].

Vitamin C, otherwise known as ascorbic acid, is a hydrophilic vitamin which has hydroxyl groups at a double bond in a lactone ring. This allows the vitamin to be a donor of protons and electrons, which is critical in its ability to reduce ROS, including superoxide anions, hydroxyl radicals, and singlet oxygen (Figure 4) [92]. Additionally, vitamin C may prevent cancer by modulating different biological processes. Vitamin C is a critical cofactor for many groups of hydroxylases that are involved in regulating the transcription factor hypoxia-inducible factor 1 (HIF1) [92]. Elevated HIF activity can foster the stem cell phenotype, making the cancer more lethal due to the tumor cell’s ability to rapidly divide and promote poor blood vessel development. In order to control HIF and prevent tumor development, HIF hydroxylases must tag the protein for degradation. Vitamin C functions as a cofactor for the HIF hydroxylases; therefore, when cells are deficient in vitamin C acid, HIF hydroxylase activity decreases and HIF transcription activity is increased [92]. When HIF levels are high there is increased tumor growth and development, but with the opposing hydroxylases present, HIF can be managed to prevent tumorigenesis [92]. Thus, vitamin C is critical for these hydroxylases to function, supporting its possible role in cancer prevention. This has led to the growing research evaluating the addition of vitamin C acid to cancer cells to decrease proliferation [92]. While it is possible that the anti-cancer effect of vitamin C may be attributed to its role in modulating HIF function, there may be multiple pathways by which this effect occurs.

There are a number of studies demonstrating vitamin C’s antioxidant properties. In a study conducted by Lutsenko et al., human kidney 293T cells were treated with 100 micromolar vitamin C and 0.2 micromolar Ci of L-[14C]ascorbic acid for vitamin C uptake or to a mixture of vitamin C and ascorbate oxidase for dehydroascorbic acid uptake. The cells were lysed, and DNA was digested and analyzed for oxidative damage. Cells that were incubated with 100 micromolar of copper and 5 mM H_2_O_2_ had significant oxidative damage (*p* < 0.5). Cells that were incubated with the copper and H_2_O_2_ then with 500 micromolar radiolabeled vitamin C or dehydroascorbic acid showed a decrease in oxidative DNA damage in normal and glutathione depleted cells [94]. Overall, when the cells were exposed to the vitamin C, the DNA exhibited less oxidative damage compared to the control. This study provides support to vitamin C acting as an antioxidant to prevent oxidative damage, which may reduce tumorigenesis. Leekha et al. tested vitamin C and its anticancer properties with cisplatin chemotherapy on SiHa and HEK293, which are cervical cell lines and control cell lines, respectively. They analyzed the cytotoxicity in cervical cancer cells at varying concentrations of cisplatin and vitamin C separately and combined. Dosing ranged from 5 to 200 micromolar for cisplatin and 25, 50, and 100 μg/mL of vitamin C for 24, 48, and 72 h. The MTT assay used combinations of 100 micromolar cisplatin + 100 μg/mL vitamin C, 50 micromolar cisplatin + 100 μg/mL vitamin C, 5 micromolar cisplatin + 100 μg/mL vitamin C, 1 micromolar cisplatin + 100 μg/mL vitamin C, and 50 micromolar cisplatin + 50 μg/mL vitamin C for time periods 24, 48, and 72 h. There was no significant difference in cytotoxicity across all doses and time periods for the HEK293 cell line, which was the non-tumor control cell line from embryonic kidney stem cells. However, there was a significant difference across each time period and varying doses on the SiHa cell lines, which are the cervical cancer cell lines. The combination of vitamin C and cisplatin showed a synergistic amplification in cell death against the cervical cancer cell line SiHa [95]. This means that vitamin C is selective for cancer cells and enhanced the killing of tumor cells.

### 4.2. Vitamin C Has Pro-Oxidant and Gene Regulator Properties

While there is a growing body of evidence demonstrating that vitamin C acts as an antioxidant, it also may contain pro-oxidant functions that lead to cellular damage in vitro. The pro-oxidant features of vitamin C are emphasized when it interacts with metals, such as iron and copper. Here, vitamin C will act as a reducing agent and then form oxygen free radicals [93]. Interestingly, one mechanism by which vitamin C reduces tumorigenesis may be related to these pro-oxidant capacities. Chen and colleagues evaluated whether pharmacologic doses of vitamin C would reduce tumor growth in mice with aggressive glioblastoma, pancreatic, and ovarian tumor xenografts [96]. They discovered that vitamin C supplementation led to an increase in vitamin C radical and hydrogen peroxide formation and a decrease in tumor size across all tumor types by 41–53%. This occurred in the interstitial fluid of tumors and not in the blood, suggesting a targeted effect with potentially minimal side effects. Another study evaluated the cytotoxicity of ascorbate, with similar results. Ascorbate was shown to induce apoptosis due to the extracellular generation of hydrogen peroxide [97]. Given the targeted impact of ascorbate on cancer cells, there is some rationale that this pro-apoptotic effect may occur in newly initiated cancer cells, preventing their proliferation and tumorigenesis.

Additionally, vitamin C may indirectly decrease tumorigenesis via its actions as a cofactor for enzymatic reactions. Peng and colleagues evaluated the role of vitamin C in the transition of 5-hydroxymethlcytosine (5hmC) to 5-methylcytosine (5mC), a methylated form of the DNA base cytosine [98]. Loss of 5hmC, which corresponds with increasing DNA methylation, is considered to be an important marker of tumorigenesis [99]. Vitamin C acts as a cofactor for Fe-2-oxoglutarate dioxygenases, which include ten-eleven translocation (TET) enzymes [100]. TETs reduce DNA methylation by converting 5mC back to 5hMC. Their results demonstrated that vitamin C can increase the content of 5hMC of bladder cancer both in vitro and in vivo, decreasing the malignant phenotype and thus cancer risk [101]. Additionally, ascorbate has been shown to accumulate intracellularly and promote TET activity in hematopoietic stem cells, decreasing leukemogenesis [102]. Similar results have been demonstrated in melanoma cells [103]. Further, vitamin C may inhibit tumorigenesis via mitochondrial dysregulation [104]. In pancreatic adenocarcinoma cell lines, vitamin C supplementation resulted in decreased cell growth via the inhibition of glucose metabolism without altering the levels of ROS [104]. The mechanism by which this occurs is largely unknown but is believed to be related to mitochondrial dysregulation because the addition of pyruvate to the medium rescued cancer cells from death. This suggests that vitamin C supplementation may decrease pyruvate concentrations, suppressing cellular respiration.

### 4.3. Vitamin C: Clinical Evidence

Numerous in vitro studies demonstrate vitamin C’s ability to prevent oxidative stress in human cell lines, a process which has also been shown to occur in the human body. Cooke and colleagues measured urinary and serum levels of 8-oxo-2′-deoxyguanosine (8-oxodG) to evaluate oxidative stress [105]. They measured serum and urinary 8-oxodG after the supplementation of 500 mg of vitamin C in both experimental and control subjects over the course of 25 weeks. Vitamin C supplementation began 3 weeks after a baseline of 8-oxodG was established. After the vitamin C washout period, where no vitamin C was supplemented, there was a significant increase in the levels of 8-oxodG in DNA, enforcing its antioxidant effects [105]. These results were negatively correlated (Pearson r = −0.40, *p* < 0.001), but the authors did not report the experimental or control 8-oxodG levels in DNA [105]. This study was performed with only 30 healthy volunteers, making it difficult to generalize; however, other studies have shown similar results. Fraga et al. illustrated that with a decrease in the intake of vitamin C, there were elevated levels of 8-oxo-dG in human sperm [106]. In another study, 14 healthy human volunteers who had taken vitamin C had a decrease in H_2_O_2_ damage in isolated white blood cells [107]. However, there was no change in endogenous DNA damage. Brennan et al., had their participants take 1000 mg vitamin C daily for 42 days or 800 mg vitamin E for 42 days. Peripheral blood was taken and treated with 200 micromolar H_2_O_2_, 10 micromolar H_2_O_2_, or used as a control. They analyzed DNA damage using ELISA after a 3-week and 6-week wash out period. Cells that were treated with 200 micromolar H_2_O_2_ showed a significantly decreased DNA oxidative damage when supplementing with vitamin C (*p* < 0.05) [107]. For vitamin C, the %ssDNA decreased from roughly 78% to 45%. The control did not have hydrogen peroxide added nor did it have vitamins added. The DNA damage was consistent between 10 and 20% [107]. Another study examined lung cancer prevention, demonstrating that smokers who supplemented their diet with vitamin C had less oxidative DNA damage than prior to supplementation [108]. The researchers obtained results comparing 500 mg slow-release and plain release tablets of vitamin C paired with an average dose of vitamin E (91 mg), and assessed how this protocol changed the levels of endonuclease III and formamidopyrimidine DNA glycosylase enzymes, which mediate DNA repair after oxidative damage. The result was that the slow-release tablet prolonged the protective effect of oxidative DNA damage after a 4-week trial [108]. Thus, vitamin C may be a promising supplement for individuals who have a predisposition for DNA damage. These studies demonstrate that the antioxidant role of vitamin C is not limited to in vitro contexts, but occurs within the human body as well, providing some rationale for an anti-cancer effect. However, the evidence of this anti-cancer effect is unclear.

A number of observational studies have assessed dietary vitamin C intake and cancer risk, with mixed results [109]. Bo and colleagues performed a meta-analysis of the existing literature to assess the impact of dietary vitamin C on esophageal cancer risk [110]. Their meta-analysis included 15 studies, encompassing 7063 controls and 3955 cancer cases. Their results demonstrate that higher dietary vitamin C intake is inversely associated with esophageal cancer risk (overall OR = 0.58, 95% CI 0.49–0.68). Similar results were shown with bladder cancer (RR = 0.84, 95% CI 0.73–0.98) [111], breast cancer (RR = 0.89, 95% CI 0.82–0.96) [111], and prostate cancer (RR = 0.89, 95% CI 0.83–0.94) [112]. However, a number of meta-analyses demonstrate non-significant results. One meta-analysis of 47 studies found no association between dietary vitamin C intake and colorectal cancer risk (RR = 0.92, 95 % CI, 0.80–1.06) [113]. These results support the notion that vitamin C may have site-specific effects, inhibiting certain malignancies with no impact on others. Generalization of the results of these studies may be difficult due to the number of confounders that limit each study.

Additionally, many studies have evaluated the impact of supplemental vitamin C and cancer prevention. The Iowa Women’s Health Study published by Kushi et al., followed 34,387 eligible women ages 55–69 through questionnaires for four years [114]. They assessed the antioxidant vitamins A, C, and E. Women that consumed more than 10,000 IU/day of vitamin A demonstrated a slight decrease in age-adjusted risk of breast cancer (relative risk = 0.73, 95% CI 0.50–1.05). Those who took vitamin C supplements between 500 and 1000 mg/day had a relative risk of 0.79, but those who took over 1000 mg had a relative risk of 0.77 which showed insignificant differences between the two. After following the women who supplemented vitamin C, there was no significant decrease in risk of developing breast cancer and no significant protective factors against breast cancer [114]. In a case control study with 261 women with cervical cancer and 498 controls, diet was assessed and analyzed to see if there was change in cancer after the addition of different supplements. No correlation was found between vitamin C and cervical cancer risk [115]. One case control study assessed vitamin supplementation and risk of oral or pharyngeal cancer risk. After controlling for other risk factors such as smoking and alcohol, there was a significant decrease in risk when supplementing vitamin C. However, when adjusting for other use of supplements, the only vitamin that was still associated with a decreased risk was vitamin E [116]. Additionally, the PROTEUS study, which was a case-control study including 1916 patients with prostate cancer matched with 1915 controls, failed to demonstrate any relationship between dietary or supplemental vitamin C and cancer prevention [117]. Thus, the current body of evidence surrounding the supplementation of vitamin C for cancer prevention fails to demonstrate any definite conclusions. Further, if there is a benefit to supplemental vitamin C for cancer prevention, the potential mechanism may occur through a broad variety of pathways, which may or may not include its antioxidant properties. Future case-control or prospective cohort studies should be designed and control for the impact of multiple vitamin supplements in carcinogenic risk.

## 5. Vitamin E

### 5.1. Vitamin E Background and Antioxidant Properties

Vitamin E refers to a group of fat-soluble antioxidants known as tocopherols and tocotrienols. These compounds naturally occur in plants, which produce four different homologues (α-, β-, γ-, and δ-) of each depending on the placement of methyl groups on their chromanol ring. Dietarily, these nutrients are abundant in nuts, seeds, oils, and also appear in various other foods in Western diets as a supplemental additive—most commonly appearing as γ-tocopherol. The homologue α-tocopherol is found most predominantly in human tissue and appears to be preferentially absorbed and metabolized. Despite this, all forms of vitamin E share the same antioxidant mechanism, which acts by scavenging for free lipid peroxyl radicals that are biproducts of the lipid peroxidation chain reaction—particularly protecting the lipid membranes of the cell, where vitamin E is often found [40,118,119,120].

### 5.2. Vitamin E Induces Anti-Inflammatory Effects

Beyond antioxidant benefits, three variants of vitamin E—γ-tocopherol, δ-tocopherol, and γ-tocotrienol—have been found to have robust anti-inflammatory effects. Specifically, these forms of vitamin E were found to inhibit prostaglandin E2 (PGE_2_) and leukotriene B4 (LTB_4_) in epithelial cells, macrophages, and in neutrophils without directly inhibiting the enzymatic function of COX-2 and 5-LOX [118]. On the other hand, the 13′-carboxychromanol metabolite of vitamin E forms appear to inhibit 5-LOX, and the cyclooxygenase activity of COX-1 and COX-2, directly [118,121,122,123]. Additionally, γ-tocotrienol has been found to be a strong inhibitor of NF-κB activation within various cancer cell lines and lipopolysaccharide (LPS) activated macrophages. In these LPS-stimulated macrophages, the effect of limiting NF-κB activity via γ-tocotrienol decreases IL-6 and granulocyte-colony stimulating factor (G-CSF) production, hindering inflammation [118,124]. γ-tocotrienol has also been shown to inhibit JAK-STAT3 signaling in cancer cells by activating protein-tyrosine phosphatase SHP-1 [118,125]. Lastly, γ-tocotrienol can limit JAK-STAT6 signaling by blocking the phosphorylation of STAT6 and the ability of STAT6 to bind to DNA [118]. These factors have made vitamin E variants enticing as both preventative agents and as potential adjuncts to various cancer treatments. However, recent findings suggest that elevated vitamin E intake may increase the risk of various cancers, particularly prostate cancer, by inducing the expression of various cytochrome P450 enzymes [126]. In individuals such as smokers or those who work in high-risk fields, the heightened activity of these enzymes can result in an overabundance of ROS from metabolized carcinogenic compounds which displace the antioxidant effect of vitamin E, ultimately damaging DNA and other cellular components—thus increasing the risk of carcinogenesis [126].

### 5.3. Vitamin E Variants Inconsistently Performed as Cancer-Preventative Agents In Vivo

Studies investigating the application of vitamin E as a cancer-fighting supplement in vivo have prioritized utilizing the δ- and γ-tocopherol forms—likely due to recent findings suggesting that α-tocopherol is not as effective as these other tocopherols as a preventative agent and antioxidant [127,128,129,130,131,132]. One such experiment investigated the ability of a γ-tocopherol-rich mixture of tocopherols (γ-TmT; 57% γ-tocopherol, 24% δ-tocopherol, 13% α-tocopherol and 1.5% β-tocopherol) to block tumorigenesis in mice directly exposed to the carcinogen 4-(methylnitrosamino)-1-(3-pyridyl)-1-butanone (NNK) with or without the carcinogen benzo[*a*]pyrene (B[*a*]P). This was compared to the effect of γ-TmT on inhibiting tumor growth in mice xenografted with a culture of the human lung cancer cell line, H1299. Compared to mice not fed γ-TmT, which had a tumor multiplicity (the average number of tumors developed in each mouse) of 21.0, the mice exposed to NKK + B[*a*]P that received a diet containing 0.3% γ-TmT throughout the entire experiment had a tumor multiplicity of 14.8 [133]. This was a significant 30% reduction in tumor multiplicity (*p* < 0.05) [133]. This trend was also seen in NKK treated mice that received 0.3% γ-TmT supplementation only in the first three weeks of the experiment (18% reduction; *p* < 0.05), from weeks 3 to 19 (20% reduction; *p* < 0.05), and throughout the entire experiment (30% reduction; *p* < 0.05), compared to NKK treated mice not fed γ-TmT, which had a tumor multiplicity of 20.8 [133]. In mice receiving the H1299 xenograft, there was a rapid inhibitory effect of tumorigenesis observed, with a significant reduction in both tumor size (56%) and tumor weight (47%) for mice who were fed 0.3% γ-TmT compared to control mice not fed γ-TmT after the first 6 weeks [133]. Additionally, immunohistochemistry analysis to detect the cell death marker caspase 3 revealed significantly more apoptosis in samples collected from the γ-TmT-fed NKK + B[a]P mice (0.25% vs. 0.09%; *p* < 0.05) and H1299 xenograft mice (0.55% vs. 0.13%; *p* < 0.05), compared to samples from NKK + B[a]P mice and H1299 xenograft mice not supplemented with γ-TmT [133]. Tumor microvessel density (MVD) was also compared between the groups to determine the effect of γ-TmT on angiogenesis. While no significant changes in MVD were observed between H1299 xenograft groups, mice treated with NKK + B[a]P that were fed γ-TmT were found to have a significantly reduced MVD of 208 compared to non-supplemented control mice treated with NKK + B[a]P, which had an MVD of 375 (44.5% reduction; *p* < 0.05) [133]. These findings were corroborated in a study examining the ability of γ-TmT supplementation to inhibit tumorigenesis in the mammary tissue of female Sprague Dawley rats exposed to the carcinogen N-methyl-N-nitrosourea (NMU). Here, researchers discovered that 0.1%, 0.3%, and 0.5% γ-TmT diets resulted in a significant reduction in average tumor multiplicity when compared to the non-supplemented control by 40% (*p* < 0.05), 48% (*p* < 0.05), and 63% (*p* < 0.001), respectively [134]. Interestingly, the researchers noted no significant decrease in serum PGE_2_ or LTB_4_ levels with administration of the γ-TmT mixture, despite showing similar pro-apoptotic activity in the mammary tumor cells via an increased presence of caspase-3. Additionally, the supplementation of γ-TmT in these mammary tumor cells appeared to result in an increase in PPAR-γ mRNA expression, but a decrease in ER-α mRNA expression, further supporting models of nuclear receptor regulation as a mechanism for antioxidant anti-cancer activity by limiting gene expression [134].

Another similar study directly compared the ability for α-, δ-, and γ-tocopherol supplementation, as well as γ-TmT supplementation, to prevent tumorigenesis in mice with H1299 cell xenografts. Comparing tumor volumes, δ-tocopherol was found to be the most effective at limiting tumorigenesis, with γ-TmT and γ-tocopherol following close behind. Mice that were fed either 0.3% δ-tocopherol (*p* < 0.01) or 0.3 γ-TmT (*p* < 0.05) had a significantly reduced tumor weight compared to the non-supplemented control. Additionally, both 0.17% δ-tocopherol and 0.3% γ-tocopherol supplementation demonstrated a significant reduction in tumor weight compared to the non-supplemented control (*p* < 0.05). Regarding growth inhibition, tumor volume, and tumor weight, neither 0.17% nor 0.3% α-tocopherol supplementation appeared to be effective, resulting in no significant reduction compared to the non-supplemented control. In fact, 0.3% α-tocopherol supplementation resulted in barely any discernable reduction in tumor volume and weight compared to the non-supplemented control [135]. When comparing the supplementation of these different tocopherols for their ability to induce apoptosis, samples from the mice fed 0.3% δ-tocopherol showed a significant 2.7-fold increase in apoptosis compared to the non-supplemented control group (*p* < 0.01), with 0.3% γ-TmT and 0.3% γ-tocopherol displaying similarly significant yet progressively diminished apoptotic activity, respectively, while 0.3% α-tocopherol supplementation yielded no ability to initiate apoptosis [135]. The trend of α-tocopherol supplementation being ineffective at preventing tumorigenesis and inducing apoptosis compared to δ- and γ-tocopherol was also observed in a study examining the ability of the tocopherols to inhibit the development of colon cancer in rats exposed to the carcinogen, azoxymethane (AOM). In this study, 0.2% δ-tocopherol most significantly reduced measurements of carcinogenesis (aberrant crypt foci and aberrant crypt (AC) counts) per rat, decreasing each on average by 62.3% and 62.9% (*p* < 0.05), respectively, when compared to the positive control rats [129]. Furthermore, as seen in other studies comparing tocopherol efficacy, the effects of γ-tocopherol and γ-TmT displayed similar degrees of significance, although less than that of δ-tocopherol. Notably, α-tocopherol was not found to produce a significant reduction in the ACF and AC counts in supplemented rats when compared to the control group. Additionally, all tocopherols except for α-tocopherol were shown to significantly increase the level of PPAR-γ in supplemented colon cancer samples compared to the positive control group. This finding signifies their respective abilities to impact nuclear receptor signaling in vivo to act as an anti-tumorigenic agent during direct carcinogen exposure, just as previously seen with the study investigating the effect of γ-TmT on inhibiting tumorigenesis in NKK + B[*a*]P treated mice. Lastly, to further exemplify the strength of δ-tocopherol, this study found that rats given 0.2 δ-tocopherol were the only group that saw a significant reduction in high-grade dysplastic ACFs, with a 70% reduction compared to the control (*p* < 0.01) [136].

One study in particular has brought scrutiny to the efficacy of γ-tocopherol in limiting the growth of tumors in vivo. This study sought to examine the efficacy of commonly recommended dosages of lycopene, selenium, and vitamin E (specifically γ-tocopherol) supplementation in limiting the continued growth of androgen-sensitive R3327-H prostate adenocarcinomas in rats. Researchers in this study found no influence of γ-tocopherol in preventing prostate tumor growth when compared to the control population [137]. This apparent inability for vitamin E to impact tumorigenesis of prostate cancer in rats may be related to recent findings that suggest vitamin E supplementation does not improve the risk of developing prostate cancer and may in fact actually increase the risk of prostate cancer in humans via the aforementioned increase in cytochrome family activity [126,129,130,138,139].

### 5.4. Vitamin E Clinical Studies

**Figure 5:**
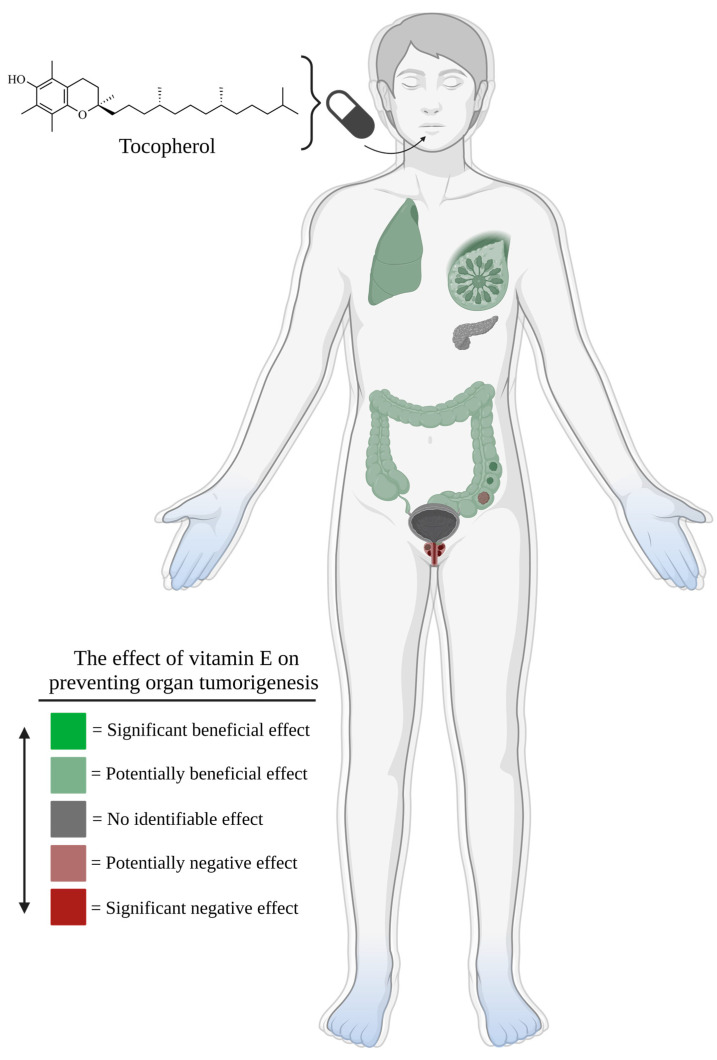
As seen in the figure scale, regions highlighted in bright green would be indicative of consistent and significant evidence supporting an inverse relationship between vitamin E supplementation and cancer risk, but no such systems exist. Pale green regions represent systems in which there is mixed evidence supporting an inverse relationship between vitamin E supplementation and cancer risk, as well as evidence supporting no relationship between vitamin E supplementation and cancer risk. Pale green regions indicate these findings for breast, lung, and colorectal tissue. Grey regions signify consistent evidence supporting no relationship between vitamin E supplementation and cancer risk. Grey regions indicate these findings for pancreatic and bladder tissue. Pale red regions represent systems in which there is mixed evidence supporting a relationship between vitamin E supplementation and increased cancer risk, as well as evidence supporting no relationship between vitamin E supplementation and cancer risk. The pale red region indicates these findings for prostatic tissue. Lastly, regions highlighted in bright red would be indicative of consistent and significant evidence supporting a relationship between vitamin E supplementation and increased cancer risk; however, no such systems exist.

As with the pre-clinical studies, clinical data regarding the role of vitamin E in cancer prevention has shown mixed results (Figure 5) [128,129,130,131,138,139,140]. One meta-analysis that primarily reviewed case-control and cohort studies regarding the usage of tocopherols as preventative agents against colorectal, lung, prostate, and breast cancer from 1986 to 2009 highlights many of the desired outcomes of vitamin E supplementation despite an equivalent presence of null findings [128]. This report identified two case-control studies and six cohort studies regarding tocopherol levels and colorectal cancer risk, with three studies citing reduced risk for tumorigenesis with increased α-tocopherol intake [128]. The remaining studies failed to identify a protective relationship between vitamin E supplementation and the risk of developing colorectal cancer [128]. Four case-control and three cohort studies were identified which discussed tocopherol levels and lung cancer risk. Of these, three case-control studies reported low serum α-tocopherol in patients with lung cancer compared to control patients, with two studies also reporting no discernable difference in serum γ-tocopherol levels between lung cancer and control patients [128]. Additionally, two of the three cohort studies claimed a significant reduction in lung cancer risk in individuals with high dietary intake of vitamin E, including in patients who had an active history of smoking [128]. For the relationship between tocopherols and prostate cancer, fourteen case-control studies and nine cohort studies were identified. Half of the case-control studies suggested that an elevated intake or serum level of tocopherols reduced the risk of prostate cancer [128]. Of these, two reported a significant inverse association between specifically serum γ-tocopherol and prostate cancer risk, instead of α-tocopherol [128]. Six of the nine cohort studies failed to identify any significant association between vitamin E consumption and prostate cancer risk; however, one study discussed the finding that strictly dietary levels of both α- and γ-tocopherol significantly reduced the risk of advanced prostate cancer [128]. Lastly, this meta-analysis identified fifteen case-control studies and nine cohort studies investigating the relationship between either serum or intake vitamin E levels and breast cancer risk. Seven case-control studies successfully reported a significant inverse association between vitamin E levels and breast cancer risk [128]. The remaining case-control studies and all of the cohort studies found no correlation between vitamin E levels and breast cancer risk [128]. Another meta-analysis investigated the associations between dietary and supplemental vitamin E intake and prostate cancer risk. From the 19 studies compiled, the estimated relative risk (RR) of developing prostate cancer from dietary levels of vitamin E intake was 0.97 (95% CI: 0.92–1.02); while, the estimated RR of developing prostate cancer from supplementary levels of vitamin E intake was 0.99 (95% CI: 0.94–1.04) [130]. Additionally, the estimated RR of developing prostate cancer from dietary and supplementary levels of vitamin E intake combined was 0.97 (95% CI: 0.85–1.08), supporting the conclusion that there was not an association between the cumulative intake of vitamin E and a change in prostate cancer risk [130].

As described previously, the Women’s Health Study was a randomized controlled clinical trial that investigated whether or not α-tocopherol supplementation diminished risks for cardiovascular events and cancer incidence in women. In this study, participants were 39,876 healthy women above the age of 45, who received either 600 IU vitamin E + aspirin placebo or vitamin E placebo + aspirin every other day for a decade [140]. Results from this study found no meaningful effect of vitamin E supplementation on breast cancer development (RR = 1.00; 95% CI: 0.90–1.12), lung cancer development (RR = 1.09; 95% CI: 0.83–1.44), colon cancer development (RR = 1.00; 95% CI: 0.77–1.31), or total cancer development (RR = 1.01; 95% CI: 0.94–1.08) in women [140]. Vitamin E supplementation also had no apparent effect on the rate of cancer death (RR = 1.12; 95% CI: 0.95–1.32) [140]. A more recent clinical trial called The Physicians’ Health Study II sought to investigate the effect of long-term vitamin E or vitamin C usage on the risk of developing various different cancers, particularly prostate cancer, in men [129]. Participants included 14,641 male physicians 50+ years old, who were given either 400 IU α-tocopherol or a placebo every other day for a decade [129]. Like in the Women’s Health Study, researchers determined that vitamin E did not convey any significant effect on the incidence of prostate cancer (HR = 0.97; 95% CI: 0.85–1.09), colorectal cancer (HR = 0.88; 95% CI: 0.64–1.19), lung cancer (HR = 0.89; 95% CI: 0.60–1.31), bladder cancer (HR = 1.21; 95% CI: 0.76–1.94), pancreatic cancer (HR = 1.14; 95% CI: 0.67–1.93), total cancer (HR = 1.04; 95% CI: 0.95–1.13), or risk of cancer death (HR = 1.13; 95% CI: 0.95–1.34) [129].

Lastly, the Selenium and Vitamin E Cancer Prevention Trial, also known as SELECT, was designed to study the impact of long-term selenium and vitamin E usage on prostate cancer risk [138]. In this study, there were 35,533 participants, each with an average risk of prostate cancer (baseline PSA of ≤4 ng/mL) [138]. Notably, African American participants were 50+ years old, while all other participants were 55+ years old. Participants either received 400 IU vitamin E + selenium placebo, 200 μg selenium + vitamin E placebo, or 400 IU vitamin E + 200 μg selenium supplementation daily for a decade [138]. Surprisingly, all treatment methods appeared to be associated with an increased risk of medium-grade prostate cancer despite none having a significant effect when compared to the placebo, with vitamin E supplementation seeing a HR = 1.16 (99% CI: 0.86–1.58) [138]. Vitamin E was also the only supplement in this study to show a significant increase in prostate cancer detection compared to the placebo with HR = 1.17 (99% CI: 1.004–1.36) [138]. Overall, clinical studies investigating a link between vitamin E and cancer development have led to mixed results, with some studies demonstrating a potential increase in cancer risk. Thus, no consensus has been reached regarding the supplementation of vitamin E and cancer prevention.

## 6. Future Directions and Conclusions

Oxidative stress has been implicated in carcinogenesis through a number of mechanisms, including lipid peroxidation, DNA damage, and its impact on both tumor suppressor genes and oncogenes. Thus, there is rationale that supplementation with antioxidants, which counter oxidative stress, may lead to a decrease in cancer risk. However, the current body of in vitro studies paint an inconsistent picture. A number of studies demonstrate evidence of this theory, but other studies fail to show any decrease in cancer formation when cell lines are treated with antioxidant supplements. Similar results were found for observational studies evaluating a link between antioxidant supplementation and cancer risk. Concerningly, some epidemiological studies even showed an increase in cancer risk in patients who used antioxidant supplements. However, these studies were limited by their inability to control for confounders or assess the temporal relationship of oxidative stress and tumor initiation. Large prospective studies following biomarkers of oxidative stress over time and investigating cancer development may address this gap in the literature. Additionally, studies examining the dysplastic transformation of different malignancies are necessary to provide more information regarding changes in oxidative biomarker levels as cancerous lesions progress. The reasoning for the lack of efficacy surrounding antioxidant supplementation as a cancer prophylactic agent is not fully elucidated but may be due to complex and incompletely characterized interactions occurring within the tumor microenvironment or elsewhere within the body. Vitamins may exert a modulatory effect on some components of the tumor microenvironment. For example, vitamin E has been shown to activate antigen-specific immune responses by dendritic cells; vitamin E has also been shown to downregulate suppression of cytotoxic T-cell activation by myeloid derived suppressor cells in leukemia [141,142]. As our understanding of these mechanisms continues to improve, the potential role of antioxidant supplementation for cancer prevention may be better characterized.

## Data Availability

No new data were created or analyzed in this study. Data sharing is not applicable to this article.
